# Her2-Functionalized Gold-Nanoshelled Magnetic Hybrid Nanoparticles: a Theranostic Agent for Dual-Modal Imaging and Photothermal Therapy of Breast Cancer

**DOI:** 10.1186/s11671-019-3053-4

**Published:** 2019-08-26

**Authors:** Qi Dong, Hong Yang, Caifeng Wan, Dongdong Zheng, Zhiguo Zhou, Shaowei Xie, Li Xu, Jing Du, Fenghua Li

**Affiliations:** 1 Department of Ultrasound, Renji Hospital, School of Medicine, Shanghai Jiao Tong University, Shanghai, 200127 China; 2 Department of Chemistry, College of Life and Environmental Science, Shanghai Normal University, Shanghai, 200234 China

**Keywords:** Theranostic agent, Anti-Her2 antibody, Ultrasound imaging, Magnetic resonance imaging, Photothermal therapy, Breast cancer

## Abstract

Targeted theranostic platform that integrates multi-modal imaging and therapeutic function is emerging as a promising strategy for earlier detection and precise treatment of cancer. Herein, we designed targeted gold-nanoshelled poly (lactic-co-glycolic acid) (PLGA) magnetic hybrid nanoparticles carrying anti-human epidermal growth factor receptor 2 (Her2) antibodies (Her2-GPH NPs) for dual-modal ultrasound (US)/magnetic resonance (MR) imaging and photothermal therapy of breast cancer. The agent was fabricated by coating gold nanoshell around PLGA nanoparticles co-loaded with perfluorooctyl bromide (PFOB) and superparamagnetic iron oxide nanoparticles (SPIOs), followed by conjugating with anti-Her2 antibodies. Cell-targeting studies demonstrated receptor-mediated specific binding of the agent to Her2-positive human breast cancer SKBR3 cells, and its binding rate was significantly higher than that of Her2-negative cells (*P* < 0.001). In vitro, the agent had capabilities for contrast-enhanced US imaging as well as *T*_2_-weighted MR imaging with a relatively high relaxivity (*r*_2_ = 441.47 mM^−1^ s^−1^). Furthermore, the Her2 functionalization of the agent prominently enhanced the US/MR molecular imaging effect of targeted cells by cell-specific binding. Live/dead cell assay and targeted photothermal cytotoxicity experiments confirmed that Her2-GPH NPs could serve as effective photoabsorbers to specifically induce SKBR3 cell death upon near-infrared laser irradiation. In summary, Her2-GPH NPs were demonstrated to be novel targeted theranostic agents with great potential to facilitate early non-invasive diagnosis and adjuvant therapy of breast cancer.

**Electronic supplementary material:**

The online version of this article (10.1186/s11671-019-3053-4) contains supplementary material, which is available to authorized users.

## Introduction

Breast cancer is the leading cause of cancer-related deaths among women worldwide [1]. The key to effectively reduce death from breast cancer is early and accurate diagnosis [2]. Despite advances in recent decades, current diagnostic and therapeutic strategies still have limitations in the early detection and precise treatment of breast cancer [3]. Therefore, it is necessary to exploit creative new strategies for breast cancer in the early and subclinical stages.

Theranostics, which involves the combination of diagnostic and therapeutic approaches within one platform, is expected to play a significant role in advancing personalized medicine [4]. Generally, theranostic agents integrate molecular imaging and therapeutic function into a single nanoparticle, given the potential to simultaneously monitor and treat breast cancer at the cellular or molecular levels [5]. In clinical, ultrasound (US) and magnetic resonance imaging (MRI) are the two commonly used methods for diagnosis and staging of breast cancer [6]. US imaging shows advantages of high safety, real-time measurement and readily availability, and contrast-enhanced ultrasound (CEUS) has improved the sensitivity and specificity in the detection of breast lesions [7]. But the application of US imaging is still bounded to relatively limited spatial and anatomical resolution. MRI could provide an image with excellent spatial resolution and soft tissue contrast, while suffering from a relatively long imaging time and limited sensitivity [8, 9]. So it is meaningful to combine the capabilities of both US and MRI to provide more complementary, synergetic, and accurate information about breast cancer [10]. In addition, surgery and adjuvant chemotherapy are the primary treatments for breast cancer, but they also carry drawbacks such as serious complications and increased multi-drug resistance. Photothermal therapy (PTT) utilizing near-infrared (NIR) lasers and photoabsorbers, has aroused widespread interest recently as an effective alternative to conventional treatments due to its minimal invasiveness and good controllability [11, 12]. Therefore, it will be of great value to combine US/MR imaging and PTT into one platform to achieve dual-modal imaging guided and monitored photothermal ablation of breast cancer.

Recently, the preparation of nanomaterials has made significant advances in the potential applications of cancer theranostics. As we all known, due to its outstanding biocompatibility and biodegradability, poly (lactic-co-glycolic acid) (PLGA)-based nanostructures have been widely investigated for use in drug-delivery systems and molecular imaging [13, 14]. Perfluorooctyl bromide (PFOB), a liquid perfluorocarbons (PFCs), has been encapsulated within polymeric shells such as PLGA to develop novel ultrasound contrast agents (UCAs) because of its high oxygen solubility, hydrophobicity, and lipophobicity [15, 16]. In addition, superparamagnetic iron oxide nanoparticles (SPIOs) have received great attention as MR molecular probes to obtain anatomical information in living organisms due to their great biocompatibility and excellent contrast enhancement [17–19].

Another promising nanoplatform is gold (Au) nanostructures, which are widely used in biological and medical researches owing to their good biocompatibility and unique optical and electrical properties [20]. The gold nanoshell, one kind of spherical gold nanomaterials, has exhibited significant surface plasma resonance (SPR) absorption in NIR region, enabling their use in PTT to selectively kill cancer cells without affecting surrounding healthy cells [21]. It has also been reported that Au nanoshells coated PLGA microcapsules could be used for US molecular imaging [22]. Numerous studies have focused on the combination of Au nanoshell and polymer as a “shell-core structure” for theranostics of cancer. Ke et al. developed a series of theranostic agents based on Au-nanoshelled poly (lactic acid) microcapsules for multi-modal imaging and PTT [23, 24]. Lu et al. prepared doxorubicin-loaded polymeric gold nanoshells for fluorescent imaging and photothermo-chemotherapy of cancer [25]. However, a common problem facing these NPs is the lack of high-affinity binding ligands on the surface, which results in their inability to recognize or bind to specific targeted cells with high specificity to maximize diagnostic and therapeutic effects. Furthermore, to the best of our knowledge, few studies have devoted to investigate targeted Au-nanoshelled PLGA hybrid nanoplatform for dual-modal collaborative diagnosis and PTT of breast cancer.

Among molecular targets considered for breast cancer so far, human epidermal growth factor receptor 2 (Her2), a cell membrane surface-bound receptor tyrosine kinase, is most commonly used as an important biomarker for the location and identification of breast cancer [26, 27]. It is overexpressed in approximately 25–30% of human primary breast cancers associated with tumor aggressiveness, high recurrence rate, and poor prognosis [28, 29]. Therefore, fabricating Her2-targeted theranostic agent is a flourishing field in improving early detection and treatment of breast cancer.

**Fig. 1:**
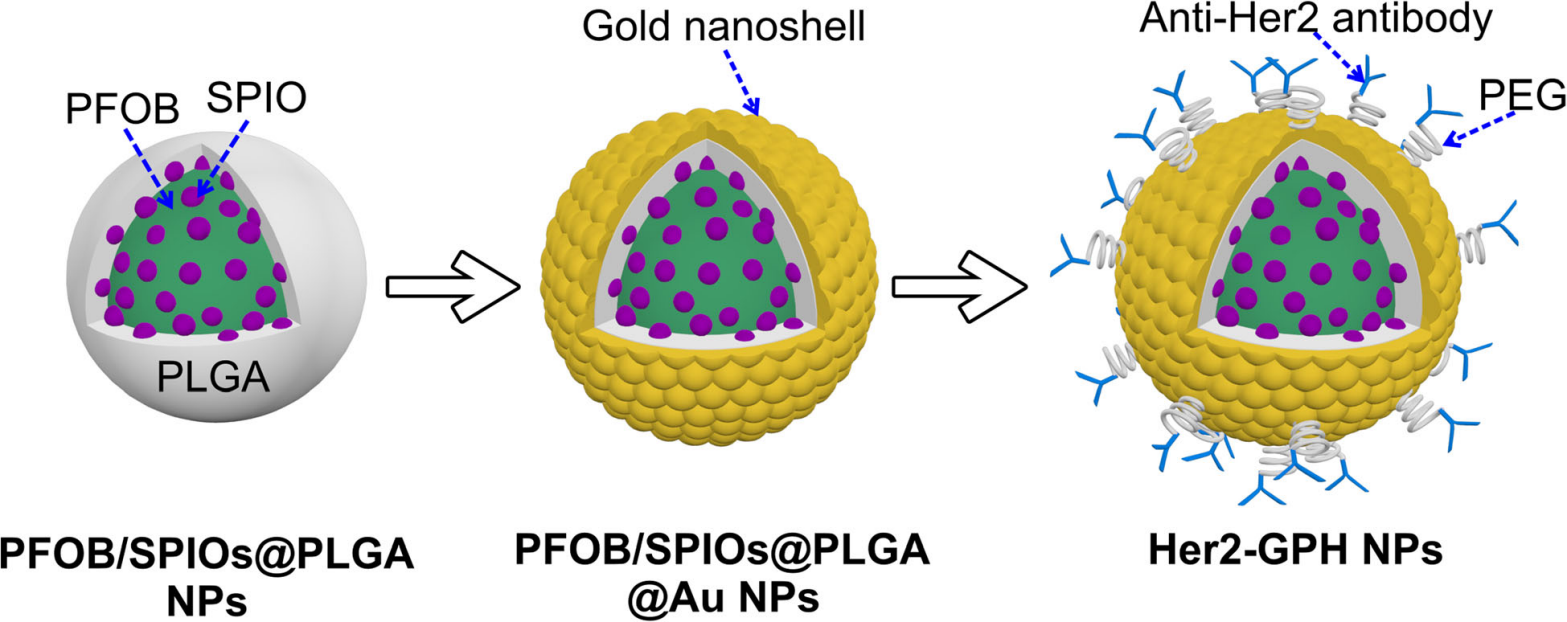
Schematic illustration of the fabrication procedure of Her2-GPH NPs

In our study, the basic idea is to develop Her2-functionalized gold-nanoshelled magnetic hybrid NPs (Her2-GPH NPs) that integrates dual-modal US/MR imaging and PTT, dedicated to the earlier diagnosis and precise treatment of breast cancer. The agent was constructed by encapsulating PFOB and SPIOs into PLGA NPs, followed by coating gold nanoshells on the surface, and then conjugation with anti-Her2 antibodies (Fig. 1). It is expected that the sufficient accumulation of the agent in Her2-positive breast cancer SKBR3 cells could be achieved by antibody-mediated targeting specificity and nanoscale size of the agent. The present study was also designed to verify the feasibility of using the agent as a dual-modal molecular probe to provide US/MR contrast-enhanced imaging in vitro, as well as the targeted PTT effect of breast cancer cells induced by NIR-absorbed Au nanoshells.

## Materials and Methods

### Materials

Poly (lactic-co-glycolic acid) (PLGA, carboxylic acid terminated, lactide: glycolide 50:50, Mw 24,000–38,000), polyallylamine hydrochloride (PAH, Mw 17,500), and tetrachloroauric (III) acid trihydrate (HAuCl_4_·3H_2_O) were obtained from Sigma-Aldrich Trading Co., Ltd. (Shanghai, China). Oleic acid-coated superparamagnetic iron oxide nanoparticles (OA-SPIOs) with a mean diameter of 10 nm were purchased from Shanghai So-Fe Biomedicine Co., Ltd. (Shanghai, China). Perfluorooctyl bromide (PFOB), polyvinyl alcohol (PVA, 87–89% mole hydrolyzed, low molecular weight), 1-(3-dime-thylaminopropyl)-3-ethylcarbodiimide hydrochloride (EDC), and *N*-hydroxysuccinimide (NHS) were obtained from Aladdin Chemistry Co., Ltd. (Shanghai, China). Carboxyl-terminated poly (ethylene glycol) (SH-PEG-COOH, Mw 2000) and fluorescein isothiocyanate (FITC)-labeled anti-Her2 antibody were obtained from Xi’an Ruixi Biological Technology Co., Ltd. (Xi’an, China) and Abcam (Cambridge, MA, USA), respectively. All other reagents used were of analytical grade.

### Preparation of PFOB/SPIOs@PLGA NPs

PFOB/SPIOs@PLGA NPs were fabricated by using an adapted oil/water emulsion solvent evaporation method [24, 30]. Briefly, oleic acid-coated SPIOs in hexane (0.5 mL, 20 mg/mL) were added to methylene chloride (3.6 mL) dissolving PLGA (100 mg) and PFOB (60 μL). Resulted organic phase was added dropwise to precooling PVA aqueous solution (20 mL, 2%, *w*/*v*). Subsequently, the system was emulsified for 2 min using probe sonication in an ice-water bath under 80% output amplitude setting. Afterwards, the emulsion was stirred at room temperature for 5 h, then centrifuged (16,000 rpm, 5 min, 15 °C, Avanti J-25, Beckman Coulter), and washed twice with deionized (DI) water to obtain PFOB/SPIOs@PLGA NPs.

### Preparation of PFOB/SPIOs@PLGA@Au NPs

Firstly, citrate-stabilized gold NPs with an average diameter of 5 nm were prepared as previously reported [21]. Meanwhile, the PFOB/SPIOs@PLGA NPs suspension (1 mL) was mixed in PAH solution (1.0 mg/mL in 0.5 mol/L NaCl aqueous solution) for 30 min, followed by centrifugation (15,000 rpm, 10 min, 15 °C) and washing twice with DI water. Then, the mixture was added in citrate-stabilized gold NPs suspension (100 ml) and stirred for 30 min. After repeated centrifuge/wash steps, the resulted Au NPs coated PFOB/SPIOs@PLGA NPs were re-dispersed into HAuCl_4_ solution (2 mL, 1% *w*/*v*) under magnetic stirring. Next, freshly prepared hydroxylamine hydrochloride solution (NH_2_OH·HCl, 0.3 mL, 0.5 mol/L) was added to reduce HAuCl_4_ to form Au nanoshells on the surface of PFOB/SPIOs@PLGA NPs.

### Preparation of Her2-GPH NPs

The prepared PFOB/SPIOs@PLGA@Au NPs aqueous solution (1 mL, 1 mg/mL) was mixed with SH-PEG-COOH (5 mg) and stirred at room temperature for 12 h. After centrifugation (16,000 rpm, 20 min, 15 °C) and washing twice, the precipitate was dispersed in phosphate buffer saline (PBS). Next, EDC (10 mg) and NHS (10 mg) were introduced and stirred at room temperature for 2 h to activate the carboxyl acid groups of the pegylated NPs, and the activated NPs (GPH NPs) were then obtained by repeated centrifugation/washing steps. Afterwards, anti-Her2 antibodies with FITC labeled (10 μL, 0.38 mg/mL) were added into the activated NPs dispersions and allowed to incubate for 90 min in an isothermal shaker. Finally, the free antibodies was removed by centrifugation/washing steps to obtain Her2 functionalized PFOB/SPIOs@PLGA@Au NPs (Her2-GPH NPs).

### Characterizations

The surface structure and morphology of Her2-GPH NPs was characterized by field emission scanning electron microscopy (FE-SEM, Hitachi S-4800, Tokyo, Japan). High resolution transmission electron microscope (TEM, JEM-2100; JEOL, Tokyo, Japan) was used to confirm the internal structure of it by dipping a sample on carbon-coated Cu grids. The corresponding energy dispersive X-ray spectroscopy (EDS) of the sample (without gold sputtering process) was attached to TEM to analyze the elemental composition of the resulted NPs. The hydrodynamic size and zeta potential of Her2-GPH NPs were measured using a dynamic laser scattering (DLS) instrument (Zetasizer Nano ZS3690; Malvern Instruments, Malvern, UK). The UV-visible absorption spectra of the agent at different preparation stages were obtained with UV-visible spectrophotometer (Beckman Coulter DU 730, USA). The amount of Au and Fe elements in Her2-GPH NPs was evaluated by inductively coupled plasma atomic emission spectroscopy (ICP-AES, Vistampxicp Varian, USA).

### Measurement of Photothermal Performance

Photothermal performance of Her2-GPH NPs was evaluated via monitoring the increase in temperature under NIR laser irradiation. Different concentrations of Her2-GPH NPs aqueous solutions (50, 100, 150, 200 μg/mL) were irradiated by an 808 nm laser (1 W/cm^2^) for 10 min in quartz cuvettes (total volume of 1 mL), and the temperature of the solutions was measured by an FLIR A300 thermal camera every 10 s. In order to assess the photothermal stability of the agent, the absorption spectrums of the materials were acquired before and after the irradiation process. DI water was irradiated under the same conditions for comparison.

### Cell Culture

Human breast carcinoma SKBR3 cell line (SKBR3 cells) overexpressing Her2 and human breast cancer MDA-MB-231 cell line (MDA-MB-231 cells) low-expressing Her2 were from the Institute of Biochemistry and Cell Biology (Shanghai, China). They were cultured in Dulbecco’s modified Eagle medium (DMEM, Gibco Life Technologies, Grand Island, NY, USA) containing 20% fetal bovine serum (FBS) and 1% penicillin-streptomycin. All the cells were grown at the environment of 37 °C and 5% CO_2_.

### In Vitro Cytotoxicity Assay

In vitro cytotoxicity of Her2-GPH NPs was evaluated by cell counting kit-8 assays (CCK-8, Dojindo Molecular Technologies Inc., Japan) on SKBR3 and MDA-MB-231 cells. The two kinds of cells were seeded respectively in 96-well plates at a density of 1 × 10^4^ per well and incubated for 24 h. After removing the culture medium, the cells were treated with different concentrations (0, 10, 20, 50, 100, 200 μg/mL) of Her2-GPH NPs and further incubated for 12 or 24 h. Then, the CCK-8 solution (10 μL CCK-8 in 100 μL DMEM) was added and incubated for an additional 2 h. Finally, the optical density (OD) of each well was measured using a microplate reader (Thermo scientific Multiskan MK3) at the wavelength of 450 nm.

### In Vitro Receptor-Specific Targeting Studies

#### Confocal Laser Scanning Microscopy

SKBR3 and MDA-MB-231 cells were plated in glass bottom cell culture dishes (*Φ* = 20 mm) at a density of 2 × 10^4^ cells/well for 24 h, and then they were divided into three groups, respectively, including non-targeted group, targeted group, and targeted inhibition group. Two kinds of cells as non-targeted group were treated with 100 μL GPH NPs, and the cells in the targeted group were treated with 100 μL Her2-GPH NPs. In targeted inhibition groups, the cells were incubated with free anti-Her2 antibodies (20 μL) for 30 min before treated with Her2-GPH NPs. After respective incubation of 30 min, the cells were washed three times with PBS, fixed with 4% paraformaldehyde for 15 min. Then the cells were stained with a nucleus staining agent (DAPI; Beyotime Biotechnology Co., Ltd., Shanghai, China) for 10 min and washed again with PBS. Finally, the cells were observed using a laser scanning confocal microscopy (LSCM) (Leica TCS SP5 II, Leica Microsystems Ltd., Mannheim, Germany).

#### Flow Cytometry

SKBR3 and MDA-MB-231 cells were cultured and treated as described above, and the groupings were consistent with LSCM assay. All the cells were digested with trypsin and then re-suspended in PBS prior to flow cytometry (FCM) with a density of 2 × 10^5^ cells per tube. The fluorescence intensity of the cells was determined by a flow cytometer (FC500MCL, Beckman Coulter, Fullerton, CA). SKBR3 and MDA-MB-231 cells were used as blank controls to set the gates. Then, the voltage and fluorescence compensation of FL1 were adjusted to ensure that the spontaneous fluorescence of cells was within 10^1^ of the fluorescence histogram. After incubating the cells with NPs, the deviations of the fluorescence intensity from the blank groups reflected the binding rate of the NPs to the cells. All experiments were carried out in triplicate.

### In Vitro US Imaging and Quantitative Analysis

#### In Vitro Solution Ultrasound Imaging

In vitro solution ultrasound imaging of Her2-GPH NPs was carried out using Mylab Twice Ultrasound System unit (Esaote SpA, Genova, Italy). Her2-GPH NPs were dispersed in degassed DI water into different concentrations (0.1, 0.5, 1, 1.5, 2 mg/mL) and injected into Eppendorf tube (2 mL), and then the tube was immerged in an ultrapure water tank. Ultrasonography was performed using LA522 transducer in both two-dimensional (2D) gray-scale mode and contrast-enhanced mode under the following parameters: the mechanical index (MI) = 0.01 and the range of frequency was 3–9 MHz. The images were acquired from the longitudinal cross-section of the tube and recorded for quantitative analysis of Time-Intensity Curve (TIC) using QontraXt V3.06 software.

#### Targeted US Imaging of Breast Cancer Cells

SKBR3 cells and MDA-MB-231 cells were cultured in 6-well plates at a density of 2 × 10^6^/well for 24 h, and they were divided into three groups, including the control group, non-targeted group, and targeted group. Cells in the non-targeted group and targeted group were treated with GPH NPs (100 μg/mL) and Her2-GPH NPs (100 μg/mL) for 30 min, respectively. Subsequently, the cells were washed with PBS, digested, and re-dispersed in test tubes with PBS (0.5 mL). Agarose gels (1%) were prepared in 20-mL glass dishes. To simulate the targeted ultrasound imaging effect of NPs on the cell surface, the cell suspensions in the six groups of test tubes were extracted separately with 1-mL syringes and slowly injected into the agar gel. Imaging was performed using a SL3116 transducer in gray-scale mode (gain = 70%; center frequency = 22 MHz; MI = 0.06). After scanning, the region of interest (ROI) in each group of ultrasound images was drawn. The gray-scale value of each ROI was quantitatively analyzed using image analysis software (DigSubAna; Shanghai Jiao Tong University, Shanghai, China) to evaluate the targeted ultrasound imaging effect of Her2-GPH NPs.

### In Vitro MR Imaging and Quantitative Analysis

#### *T*_2_ Measurement and MR Imaging of Her2-GPH NPs

MR imaging of Her2-GPH NPs and relaxivity measurements were performed using a 0.5 T systems (Shanghai Niumag Corporation ration NM120-Analyst). Her2-GPH NPs were suspended in DI water at various Fe concentrations (0, 0.005, 0.01, 0.02, 0.04, 0.08 mM), and *T*_2_-weighted MR images of the samples were acquired using spin-echo sequence (TR = 2000 ms; TE = 100 ms; slice thickness = 3 mm; FOV = 3 × 3 cm). The transverse relaxation time (*T*_2_) of water protons of the NPs aqueous solution was also measured, and the transverse relaxivity (*r*_2_) was determined through the curve fitting of 1/*T*_2_ (s^−1^) versus the concentration of Fe (mM).

#### Targeted MR Imaging of Breast Cancer Cells

The groupings and treatment of SKBR3 and MDA-MB-231 cells were consistent with targeted US imaging assay. After incubation with non-targeted GPH NPs (100 μg/mL) or targeted Her2-GPH NPs (100 μg/mL) for 30 min, the cells were washed with PBS, digested, and dispersed in xanthan gum (1 mg/mL). All samples were scanned using spin-echo sequence with the same parameters as described above. ROI of each image was defined for quantitative analysis of signal intensity with image J software.

### In Vitro Targeted PTT Evaluation

To visualize the targeted PTT effect of Her2-GPH NPs, SKBR3 cells were plated in glass bottom cell culture dishes (*Φ* = 20 mm) at a density of 1 × 10^5^ cells/well for 24 h, and they were divided into five groups respectively, including DMEM control groups with or without laser irradiation, targeted materials groups with or without laser irradiation, and non-targeted material with laser irradiation. The targeted groups were treated with DMEM dispersions containing Her2-GPH NPs (100 μg/mL) for 30 min and the non-targeted groups were treated with GPH NPs (100 μg/mL) at the same condition. Cells in laser irradiation groups were irradiated with NIR laser (808 nm, 1 W/cm^2^) for 10 min and further incubated at 37 °C for 2 h. Then, the cell viability was assessed using a live/dead cell assay kit (Life Technologies, Grand Island, NY). Finally, the images of the stained cells were acquired with a LSCM. To quantify the photothermal cytotoxicity, SKBR3 cells (1 × 10^4^ per well) were plated in 96-well plates and incubated for 24 h. The groupings were consistent with the above, and the cell viability was tested by CCK-8 assay. To further quantitatively evaluate the photothermal cytotoxicity of Her2-GPH NPs under different concentrations and laser irradiation. The cells were incubated with DMEM dispersions containing Her2-GPH NPs of different concentrations (0, 50, 100, 150, 200 μg/mL). After washing with PBS (10 mM, pH 7.0), the cells were irradiated by NIR laser (808 nm, 1 W/cm^2^) for 10 min and further incubated for 2 h. Then the cell viability was also determined by CCK-8 assay. Results are shown as mean ± standard deviation (*n* = 4).

To simulate the heterogeneous expression of Her2 in breast tumor tissues, we co-cultured SKBR3 cells and MDA-MB-231 cells in different proportions (0:100%, 25:75%, 50:50%, 75:25%, 100:0%). All the cells were treated with Her2-GPH NPs (200 μg/mL) for 30 min, and irradiated with NIR laser (808 nm, 1 W/cm^2^) for 10 min. After further incubation for 2 h, the cell viabilities were analyzed with CCK-8 assays. Results are shown as mean ± standard deviation (*n* = 4).

## Statistical Analysis

Quantitative data were expressed as the mean ± standard deviation (mean ± SD). Statistical differences among multiple groups were determined by analysis of variance (ANOVA), and data from two independent samples were compared using Student’s *t* test. *P* < 0.05 was considered statistically significant difference. Statistical analyses were performed using SPSS v17.0 (IBM, Armonk, NY, USA).

## Results and Discussions

### Characterizations

**Fig. 2:**
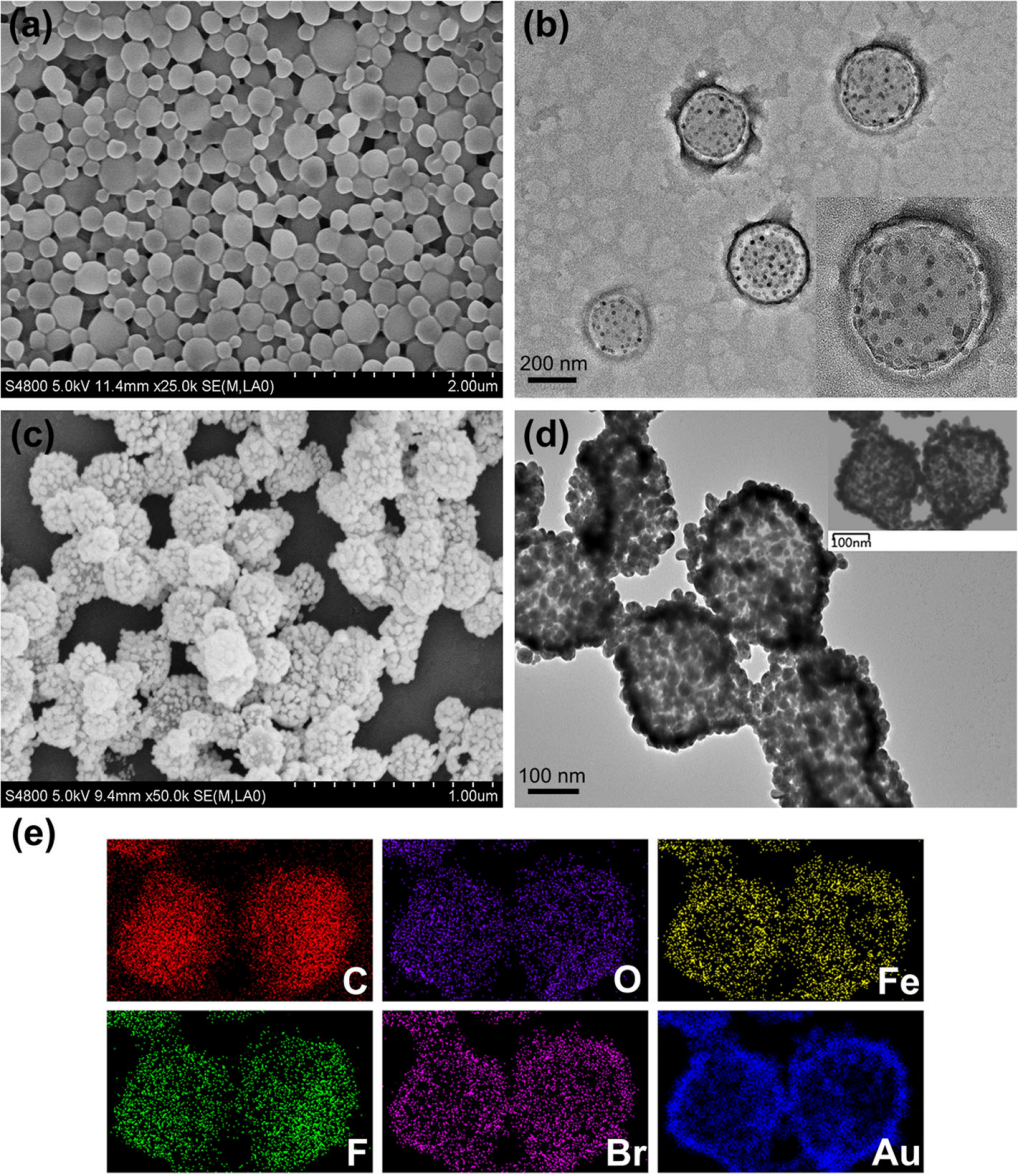
Characterizations of Her2-GPH NPs. **a** SEM (scale = 2 μm) and **b** TEM (scale = 200 nm) images of PFOB/SPIOs@PLGA NPs; **c** SEM (scale = 1 μm) and **d** TEM (scale = 100 nm) images of Her2-GPH NPs; **e** EDS element mapping images show the distributions of C, O, Fe, F, Br, and Au elements in Her2-GPH NPs

PFOB/SPIOs@PLGA NPs were fabricated via the oil/water emulsion solvent evaporation process. SEM image (Fig. 2a) revealed that the PFOB/SPIOs@PLGA NPs possessed uniform spherical morphology and smooth surface. As shown in TEM (Fig. 2b), there was an obvious difference of electronic density between the core and the shell of the PFOB/SPIOs@PLGA NPs, suggesting the encapsulation of PFOB inside the NPs. Besides, as depicted in the inset image of higher magnification, the presence of SPIOs was demonstrated as deep gray spots in the shell and liquid PFOB core region of PFOB/SPIOs@PLGA NPs. The mean diameter of PFOB/SPIOs@PLGA NPs was about 248.3 nm with a polydispersity index of 0.037, and the zeta potential was approximately − 14.7 mV according to the DLS measurement. Therefore, PFOB/SPIOs@PLGA NPs could easily absorb positively charged PAH so as to subsequently attach negatively charged Au nanoparticles with a mean size of 5–7 nm, which were used as seeds to nucleate the growth of gold nanoshell around the surface of PFOB/SPIOs@PLGA NPs through seeding procedure.

**Fig. 3:**
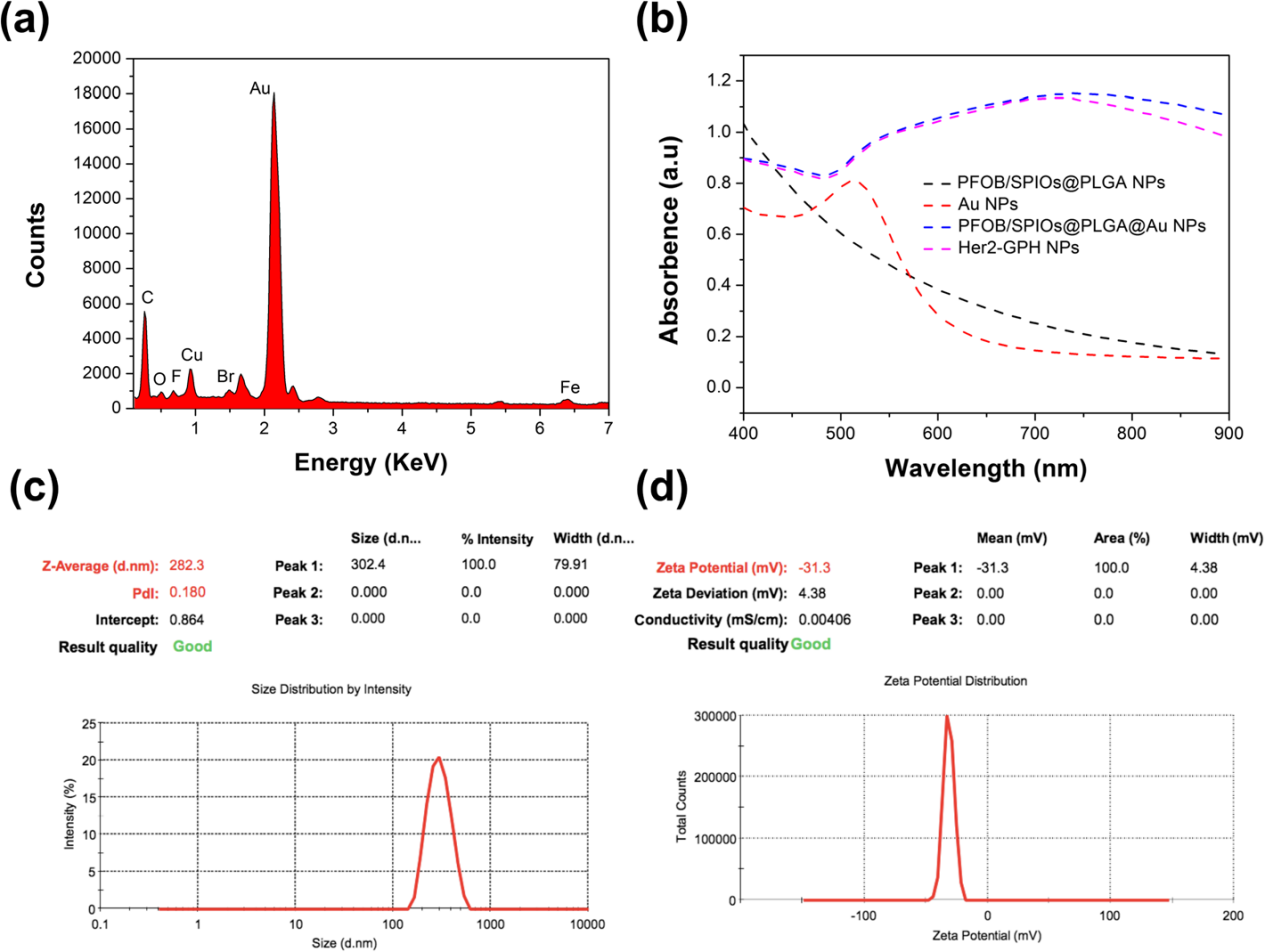
Characterizations of Her2-GPH NPs. **a** EDS elements analysis of Her2-GPH NPs; **b** UV–Vis absorption spectra of Her2-GPH NPs at different preparation stages; **c** Size distribution and **d** Zeta potential of Her2-GPH NPs

Her2-GPH NPs were prepared by linking anti-Her2 antibodies to PFOB/SPIOs@PLGA@Au NPs via SH-PEG-COOH. In this process, classical carbodiimide technology was used to activate the carboxylic acid groups of PEGylated PFOB/SPIOs@PLGA@Au NPs and promote the covalent bonding of amino groups to antibodies [31]. As illustrated in SEM and TEM images (Fig. 2c, d), Her2-GPH NPs maintained a well-defined spherical morphology with rough surface, and dense Au NPs with a diameter of tens of nanometers could be clearly seen on the surface of the NPs, which indicated the successful fabrication of the gold nanoshells. EDS elements mapping (Fig. 2e) and elements analysis results (Fig. 3a) of Her2-GPH NPs clearly revealed the large amount of Au element and the presence of Fe, F, and Br elements, indicating the successful encapsulation of SPIOs and PFOB and the formation of Au nanoshells. Moreover, the content of Au and Fe elements in Her2-GPH NPs were evaluated to be 67.71 ± 7.34% wt.% and 2.13 ± 0.52% wt.% by ICP-AES, respectively.

In addition, UV–Vis absorption spectra of Her2-GPH NPs at different preparation stages were also examined (Fig. 3b). PFOB/SPIOs@PLGA NPs showed no obvious absorption peak in the range from 400 to 800 nm while Au NPs exhibited a plasma resonance peak at about 520 nm. Both PFOB/SPIOs@PLGA@Au NPs and Her2-GPH NPs exhibited a continuous broad peak ranging from 600 to 900 nm (NIR region) because of the attached Au seeds grown larger enough through seeding process to cluster. The broad absorption spectra in NIR region ensured the Her2-GPH NPs could operate as photoabsorbers for NIR photothermal therapy. Furthermore, compared with PFOB/SPIOs@PLGA NPs, Her2-GPH NPs had an increased size distribution of 282.3 nm with a polydispersity index of 0.18 (Fig. 3c). And the zeta potential was − 31.3 mV (Fig. 3d), implying the good stability of it.

### Measurement of Photothermal Performance

**Fig. 4:**
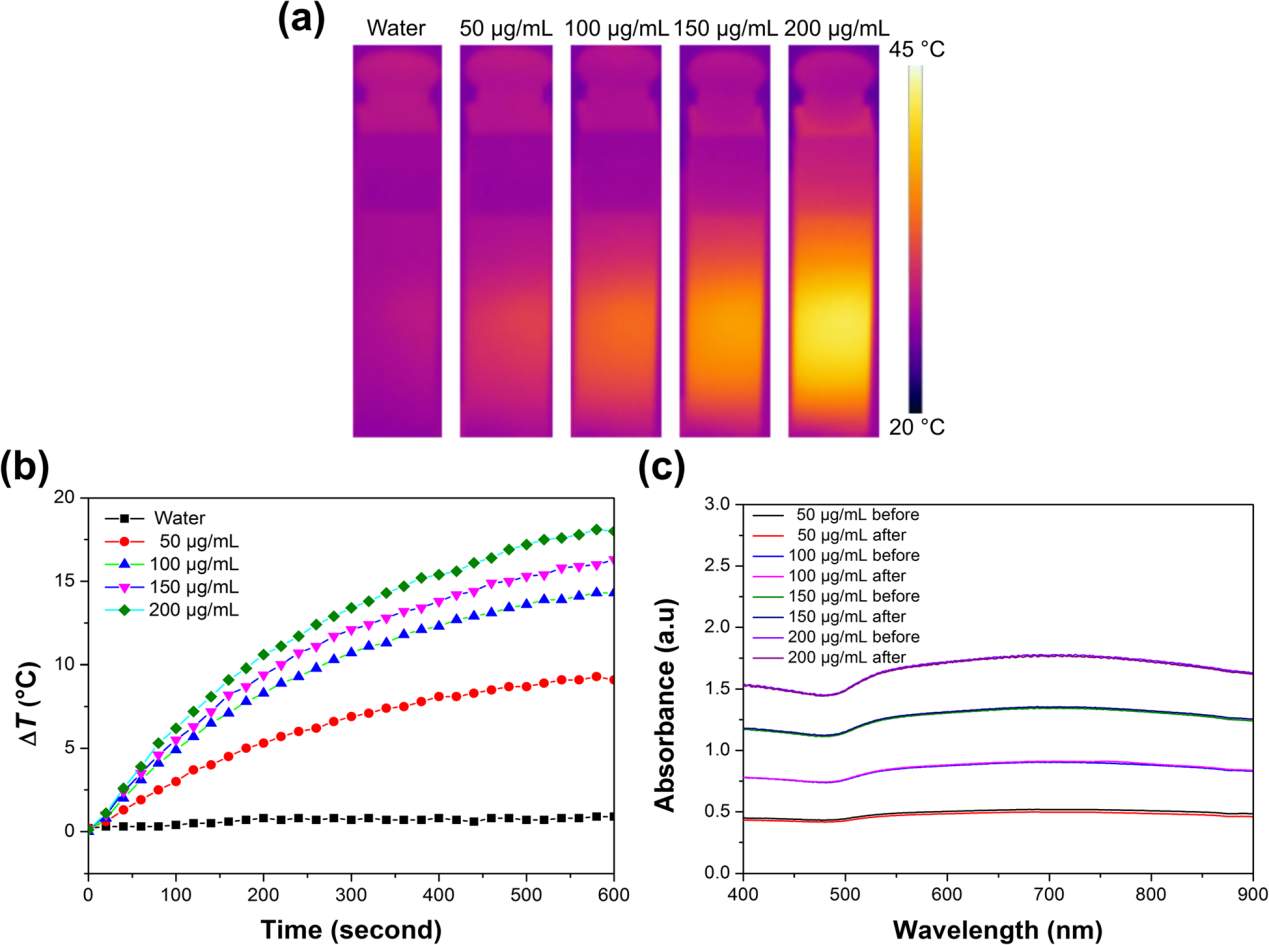
Photothermal effect of Her2-GPH NPs. **a** NIR thermal images and **b** temperature variation of Her2-GPH NPs at different concentrations, after irradiation by an NIR laser (808 nm, 1 W/cm^**2**^,10 min); **c** UV-Vis-NIR absorption spectra before and after the photothermal irradiation assay

The photothermal conversion effect of Her2-GPH NPs solution was evaluated under the irradiation of NIR laser (808 nm, 1 W/cm^2^), and temperature variation was monitored with an IR thermal-imaging camera every 10 s. After 10 min of laser irradiation, the thermal imaging color of different concentrations of Her2-GPH NPs solution changed, signifying that temperature elevated with increasing concentration of Her2-GPH NPs (Fig. 4a). Photothermal temperature measurement was consistent with the imaging data. It could be observed that the temperature of the NPs solution rose with increasing exposure time and concentration (Fig. 4b). In detail, the temperature increase of Her2-GPH NPs solution was 18.5 °C at the concentration of 0.2 mg/mL, while there was only 1.2 °C increase for DI water. In order to kill cancer cells, the temperature had to be raised to the hyperthermia temperature range (40–47 °C) according to previous studies [32]. However, due to the thermal loss in vitro, a temperature increase of about 10 °C is not sufficient to induce cell death [33]. Her2-GPH NPs could increase the temperature by about 18 °C at relatively low concentration, thus giving the potential to kill cancer cells by hyperthermia. Furthermore, to verify the photothermal stability of Her2-GPH NPs, UV-visible NIR absorption spectra of samples were collected before and after laser irradiation. And the absorption spectra are almost unchanged before and after laser exposure (Fig. 4c), ensuring Her2-GPH NPs could be served as efficient photoabsorber for PTT of cancer.

#### In Vitro Cytotoxicity Assay

**Fig. 5:**
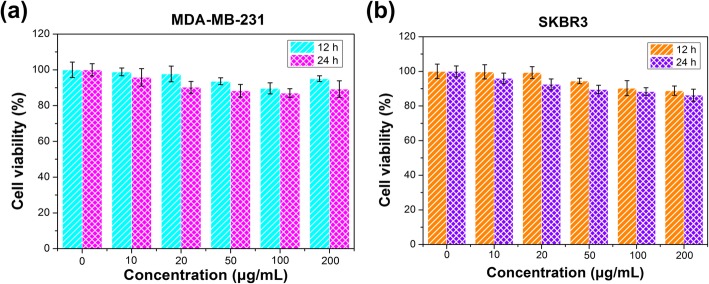
Cell viabilities of **a** MDA-MB-231 and **b** SKBR3 cells at different dosages of Her2-GPH NPs (0, 10, 20, 50, 100, 200 μg/mL) for 12 h and 24 h (data expressed as mean ± SD, *n* = 4)

It is well known that biocompatibility of NPs is one of the primary concerns in biomedical applications and should be determined first. Thus, CCK-8 assays were used to evaluate the cytotoxicity of Her2-GPH NPs in breast cancer MDA-MB-231 and SKBR3 cells, as shown in Fig. 5a and b. Compared with controls, the viability of Her2-GPH NPs-treated MDA-MB-231 and SKBR3 cells remained more than 90% at the concentration ranging from 10 to 200 μg/mL for 24 h, indicating the low cytotoxicity and good biocompatibility of the resulted NPs, which could ensure their safety for further cell experiments and clinical studies.

### In Vitro Receptor-Specific Targeting Studies

#### Confocal Laser Scanning Microscopy

**Fig. 6:**
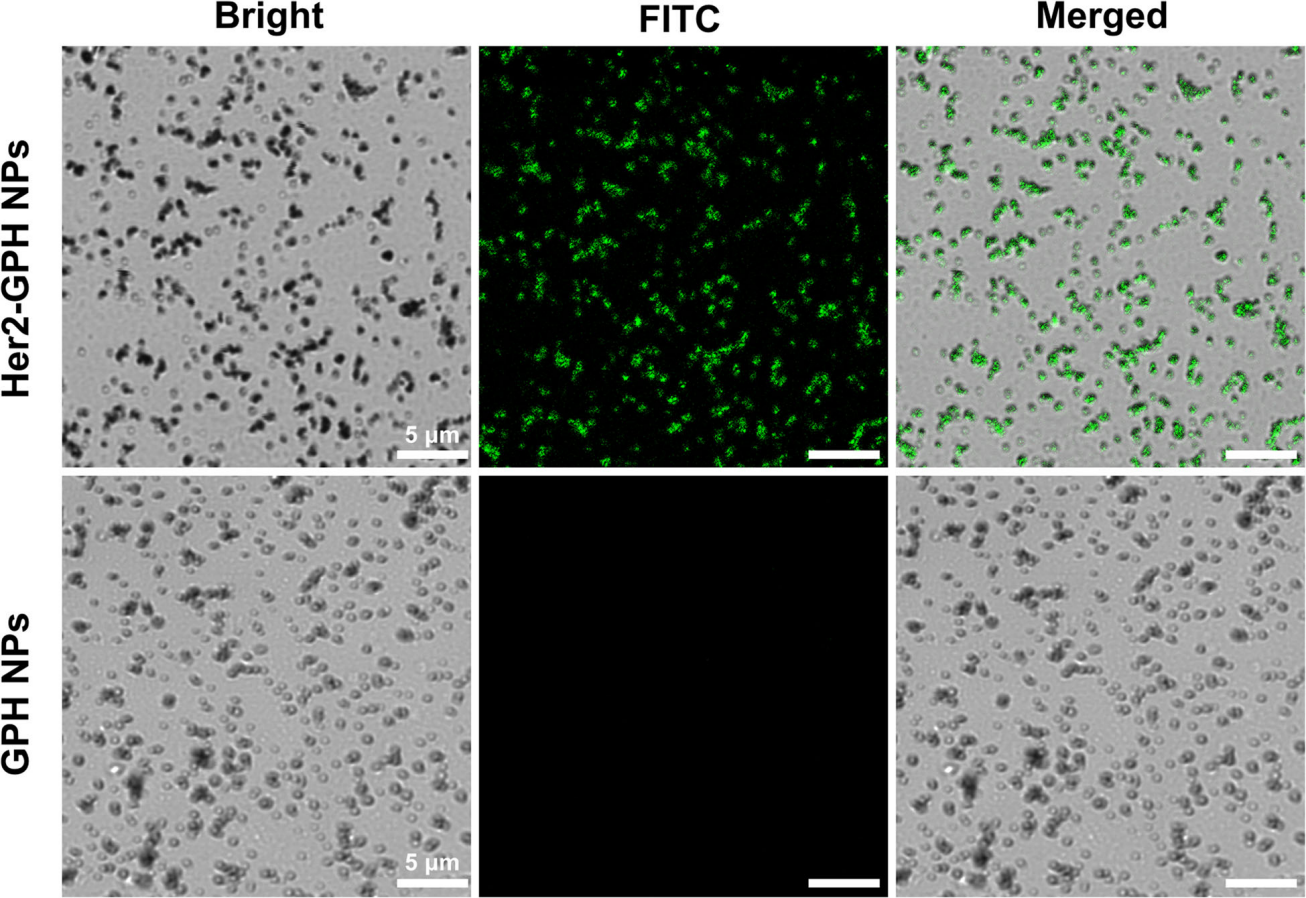
Confocal microscopic images of Her2-GPH NPs and GPH NPs in bright field, FITC fluorescence and merged channels (scale bar = 5 μm)

Fluorescein isothiocyanate (FITC) is widely used as green fluorescence probes for immune detection and it can easily bind several kinds of monoclonal antibodies. Thus, we choose FITC-labeled anti-Her2 antibody, and the connection of NPs with antibody could be observed more visually by immunofluorescence assay. To verify the binding of anti-her2 antibodies to GPH NPs, GPH NPs and Her2-GPH NPs were placed on dishes (*Φ* = 20 mm) for CLSM analysis. As illustrated in Fig. 6, both Her2-GPH NPs and GPH NPs could be clearly observed in bright field. GPH NPs showed no fluorescence signal, while Her2-GPH NPs exhibited bright green fluorescence in the FITC and merged channel, which confirmed the successful conjugation of anti-her2 antibody with GPH NPs.

**Fig. 7:**
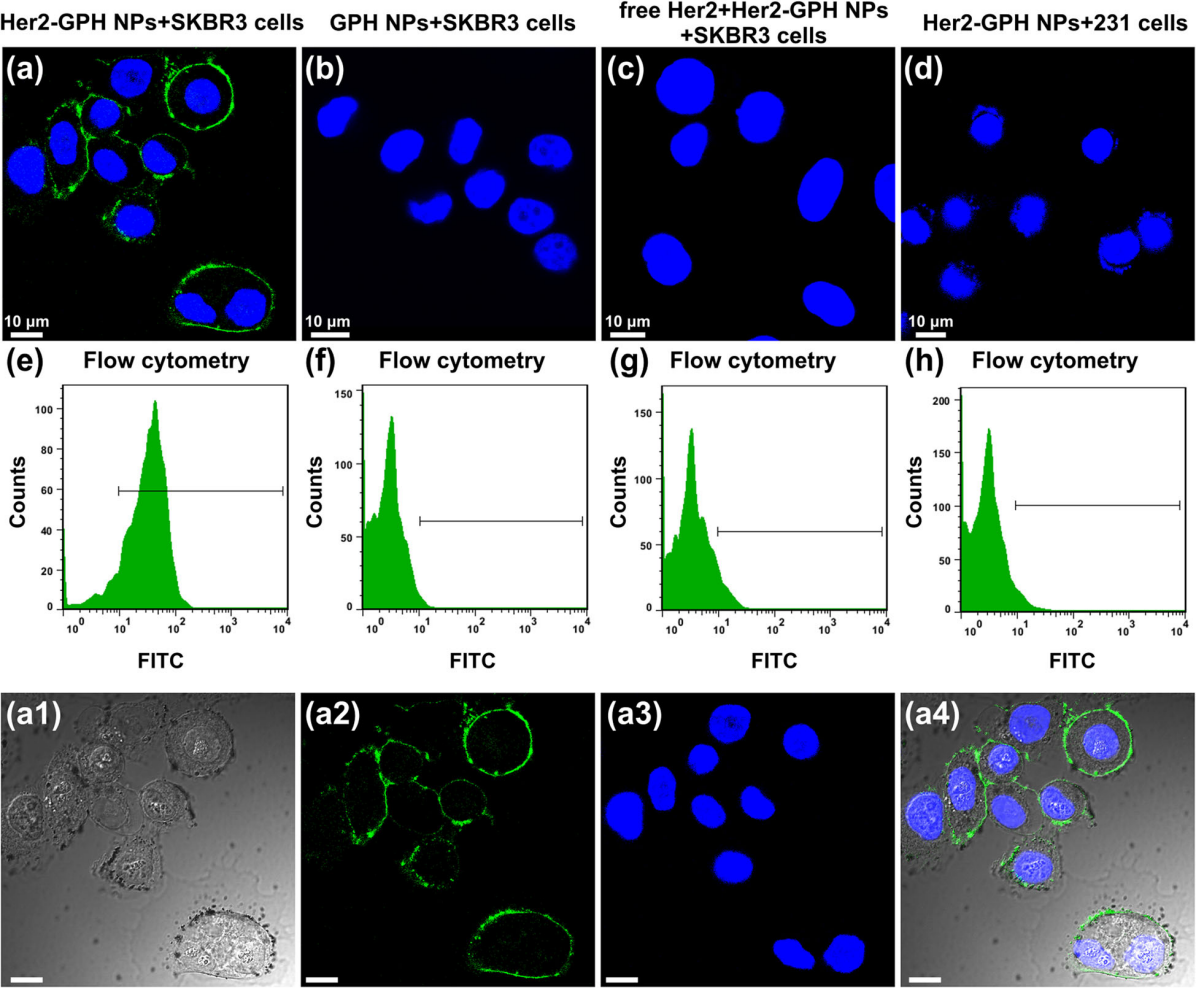
Confocal microscopic images and flow cytometry results of SKBR3 cells incubated with targeted Her2-GPH NPs (**a**/**a1**–**a4**, **e**) and non-targeted GPH NPs (**b**, **f**), free Her2 antibody pre-treated SKBR3 cells incubated with Her2-GPH NPs (**c**, **g**), and MDA-MB-231 cells incubated with Her2-GPH NPs (**d**, **h**). **a1** the image of bright field; **a2** the image of DAPI channel; **a3** the image of FITC channel; and **a4** the image of merged channel (scale bar = 10 μm)

We utilized LSCM to confirm the targeting specificity of Her2-GPH NPs in vitro. As can be seen from the Fig. 7, compared with non-targeted group (b), SKBR3 cells incubated with targeted Her2-GPH NPs (a) exhibited bright green fluorescence signals on the surface of cell membranes, indicating the NPs carrying Her2 antibodies could effectively bind to Her2-positive cells [34]. To clearly observe the distributions of Her2-GPH NPs in SKBR3 cells, we provided a bright image (a1), FITC fluorescence image (a2), nuclear image (a3), and an overlay image (a4) of it. Because of the short incubation time of NPs, they were mainly distributed on the surface of cell membranes and aggregated into black spots. In competition study, there were negligible fluorescence signals when SKBR3 cells were pre-treated with excess free anti Her2 antibodies and then incubated with Her2-GPH NPs (Fig. 7c). These results demonstrated that the targeting behavior of Her2-GPH NPs was receptor-mediated and could be blocked by excess free anti-Her2 antibodies [26]. In addition, little fluorescence is detected in MDA-MB-231 cells treated with Her2-GPH NPs under the same conditions (Fig. 7d), which confirmed that Her2-GPH NPs specifically bind to SKBR3 cells overexpressing Her2 receptors.

#### Flow Cytometry

The binding rate of Her2-GPH NPs towards cells was further assessed quantitatively via FCM. As clearly illustrated in Fig. 7, the binding rate of targeted NPs to SKBR3 cells (e) was remarkably higher than that of MDA-MB-231 cells (h) (86% ± 3.72% vs 2.31% ± 0.36%, *P* < 0.001), indicating the specific targeting capability of Her2-GPH NPs to Her2-positive cells. In addition, Her2-GPH NPs had little targeting binding to SKBR3 cells which were pre-treated with excess free anti-Her2 antibodies (Fig. 7g), and there was significant difference between the targeted inhibition group and targeted group (3.16% ± 0.41% vs 86% ± 3.72%, *P* < 0.001). Based on the above, the FCM results provided further strong evidence that Her2-GPH NPs had specific targeting capability of recognizing and combining with SKBR3 cells overexpressing Her2 receptors and the targeting behavior was receptor-mediated.

### In Vitro US Imaging and Quantitative Analysis

#### In Vitro Solution Ultrasound Imaging

**Fig. 8:**
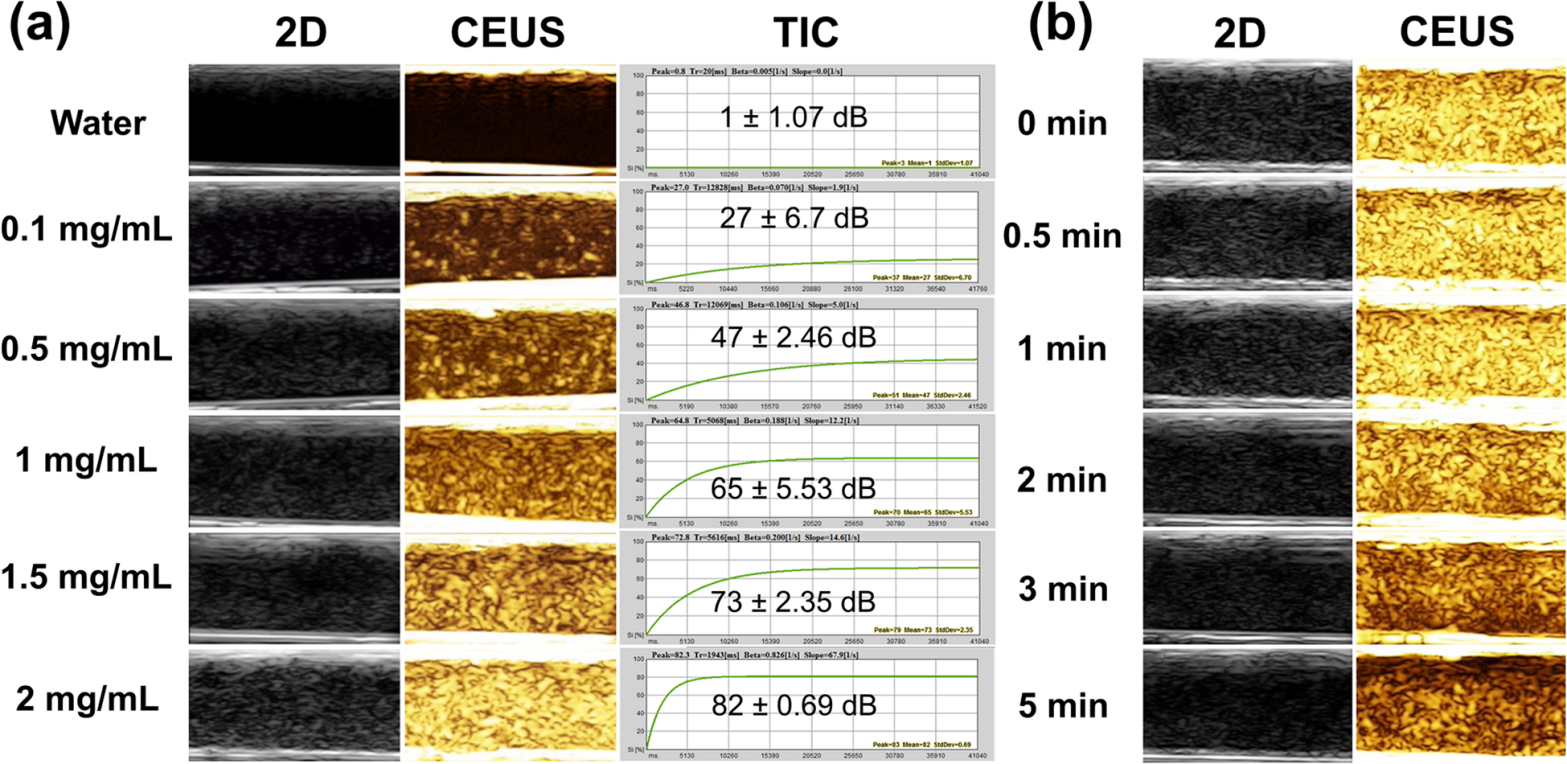
**a** In vitro two-dimensional (2D) gray-scale ultrasound (US) and contrast-enhanced ultrasound (CEUS) images and the time-intensity curve (TIC) of Her2-GPH NPs under different concentrations (0, 0.1, 1, 1.5, 2 mg/mL). **b** In vitro US images of Her2-GPH NPs (2 mg/mL) with time elapsing

In our study, the US imaging effect of Her2-GPH NPs solution was assessed in vitro under different modes using Mylab Twice Ultrasound System unit. Observed from Fig. 8a, the tubes filled with Her2-GPH NPs solution displayed strong dotted echo in both 2D gray-scale and CEUS modes. By contrast, DI water exhibited as an echo. Furthermore, with the increasing concentration of Her2-GPH NPs, the ultrasound contrast enhancement signal gradually increased. As illustrated in TIC, the mean signal intensity of the Her2-GPH NPs gradually increased as the concentration of NPs increased and it kept correspondence with the CEUS result. The agent at the concentration of 2 mg/mL had significantly stronger contrast enhancement signals than the NPs at 0.1 mg/mL (82 ± 0.69 dB vs 27 ± 6.7 dB, *P* < 0.01), indicating the stronger backscattering is produced by higher concentration of the NPs. The above indicated that the Her2-GPH NPs possessed satisfactory contrast enhancement effect in vitro and such contrast enhancement was concentration-dependent. In addition, the length of ultrasound imaging time of Her2-GPH NPs at the concentration of 2 mg/mL was also investigated (Fig. 8b). Her2-GPH NPs exhibited exquisite contrast-enhanced signal before 2 min, and the signal intensity gradually decreased with time elapsing. However, there was still obvious enhancement signal within 5 min, which can meet the requirement of clinical contrast-enhanced ultrasonography. Conventional gas-filled microbubbles as UCAs could enhance the backscattering signal of blood by non-linear oscillations under ultrasound but are not suitable for tissue molecular evaluation due to its limitation in intravascular imaging and poor stability [35]. Compared with gas-filled agent, the liquid fluorocarbon-filled PLGA nanocapsules have acoustic stability with prolonged circulation half-time [16]. Besides, our previous research found that Au nanoshells have good reflection properties because of its high atomic number and density [36]. Therefore, we combined the ultrasonic resonance properties of PFOB-filled PLGA nanocapsules with the great echogenicity of Au nanoshells, which prominently enhanced the backscattering signal of Her2-GPH NPs in vitro.

#### Targeted US Imaging of Breast Cancer Cells

**Fig. 9:**
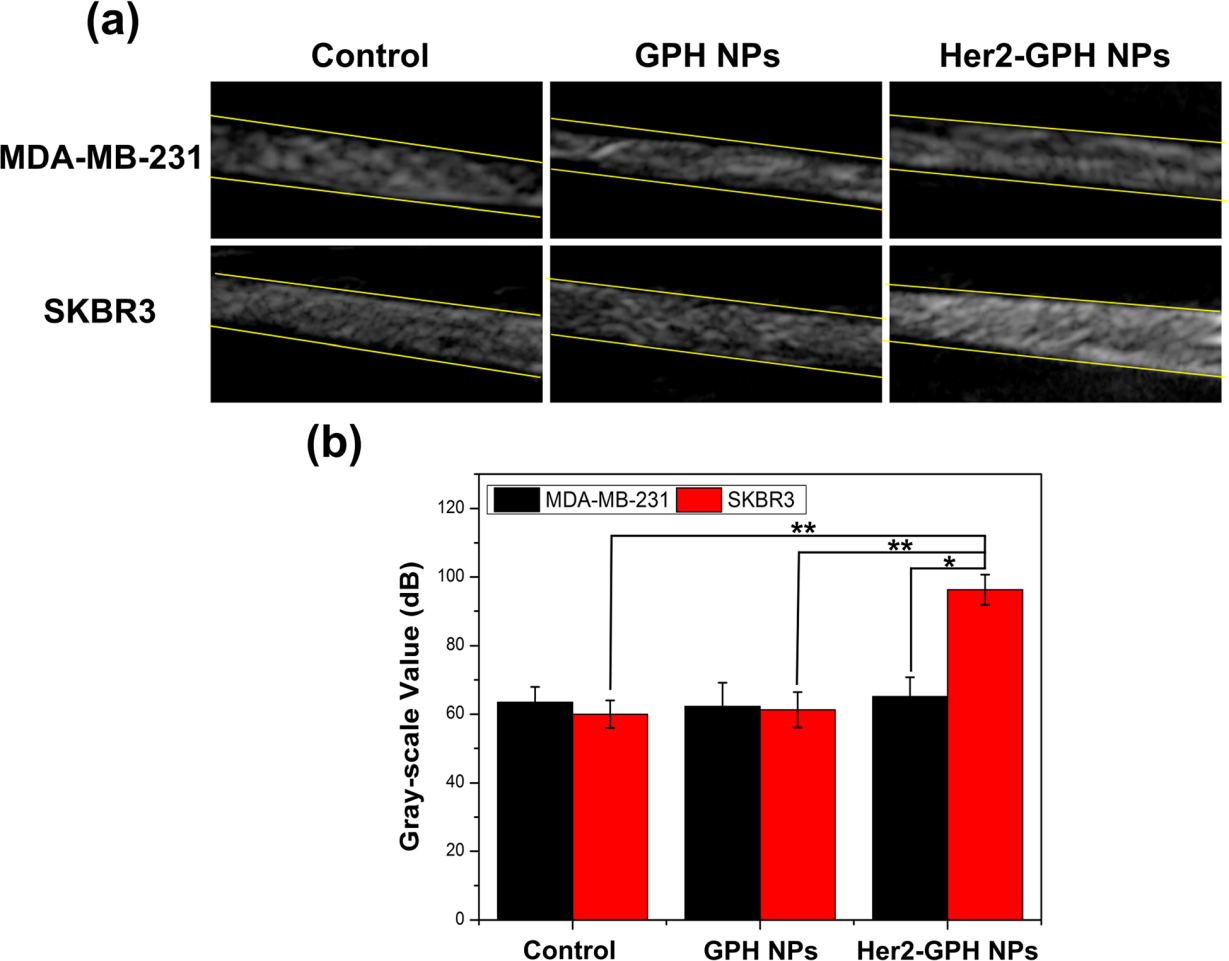
**a** In vitro ultrasound imaging and **b** gray-scale value analysis of MDA-MB-231 and SKBR3 cells treated with targeted Her2-GPH NPs or non-targeted GPH NPs. MDA-MB-231 and SKBR3 cells served as controls (data expressed as mean ± SD, *P < 0.05, **P < 0.01, *n* = 3)

The targeted ultrasound imaging ability of Her2-GPH NPs on breast cancer cells was evaluated in 2D gray-scale mode using Mylab Twice Ultrasound System unit. As shown in Fig. 9a, both the NPs-treated and the control cells displayed uniform, hyperechoic bands in the gel with clear boundaries. It could be clearly observed that the echogenic band of SKBR3 cells treated with Her2-GPH NPs was brighter and denser than those of non-targeted and control SKBR3 cells. However, no significant echo signal differences were observed in MDA-MB-231 cells treated with targeted or non-targeted NPs. We further quantified the average gray-scale value of the images based on the defined ROI, and the results were exhibited in Fig. 9b. The gray-scale value of SKBR3 cells treated with targeted Her2-GPH NPs was significantly higher than that of SKBR3 non-targeted and control group (*P* < 0.01). Moreover, SKBR3 cells treated with targeted Her2-GPH NPs showed higher gray-scale values compared to MDA-MB-231 cells treated with the same NPs (*P* < 0.05). These results demonstrated that Her2-GPH NPs could specifically bind to Her2-positive SKBR3 cells and had the potential to enhance the ultrasound molecular imaging effect of targeted cells.

### In Vitro MR Imaging and Quantitative Analysis

#### *T*_2_ Measurement and MR Imaging of Her2-GPH NPs

**Fig. 10:**
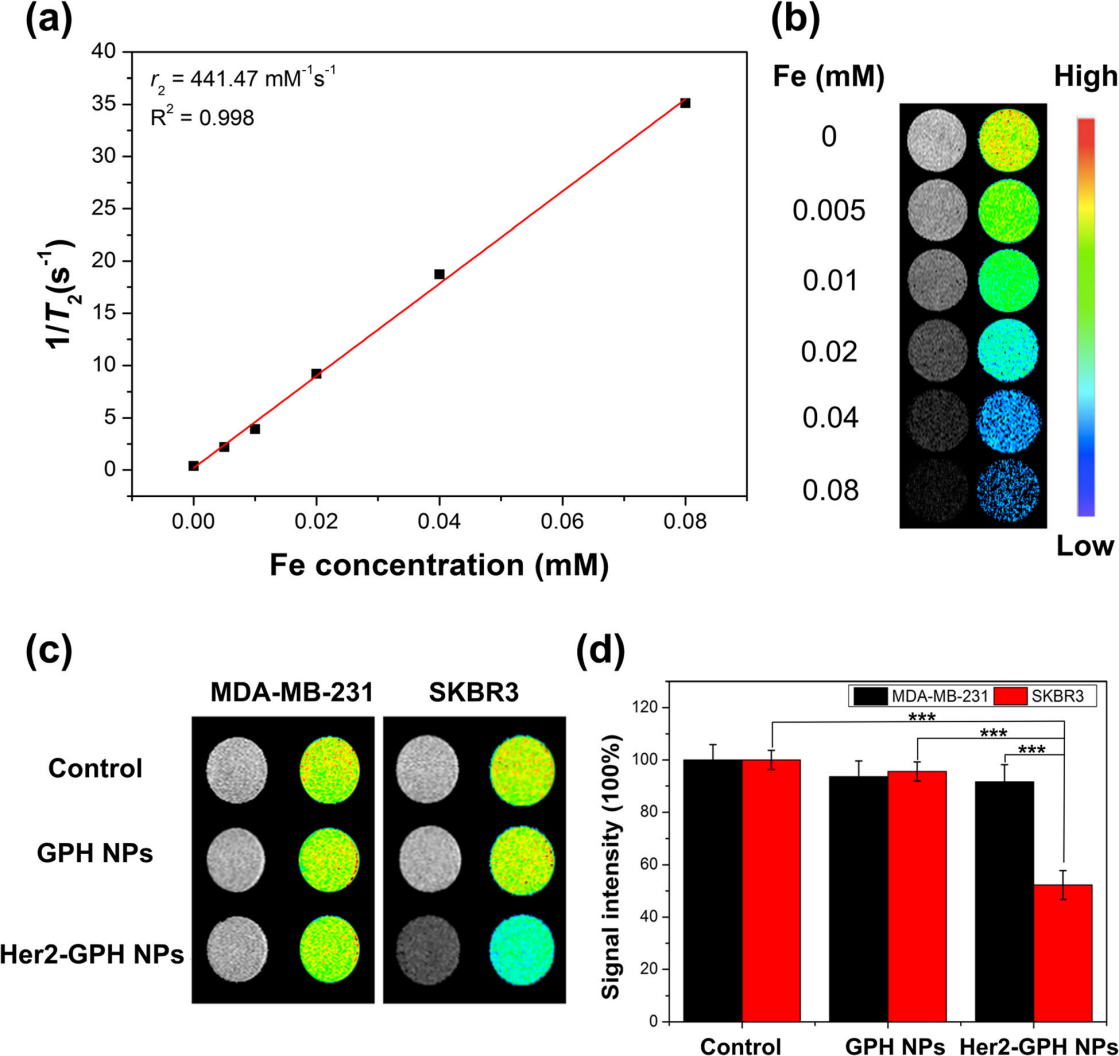
In vitro MR imaging. **a** The transverse relaxation rates (1/*T*_2_) of Her2-GPH NPs aqueous solution as a function of the iron concentration in a 0.5 T magnetic field. **b** *T*_2_-weighted MR images of Her2-GPH NPs with different iron concentrations acquired using spin-echo sequence. **c** *T*_2_-weighted MR images and **d** signal intensity of Her2-GPH NPs (100 μg/mL) and GPH NPs (100 μg/mL) incubated with MDA-MB-231 and SKBR3 cells. MDA-MB-231 and SKBR3 cells served as controls (data expressed as mean ± SD, *P < 0.05, **P < 0.01, ***P < 0.001, *n* = 3)

SPIOs have been intensively investigated in *T*_2_-weighted MR imaging due to their capability to shorten transverse relaxation times of the surrounding water protons, which results in the decrease in the MR signal intensity and enables to provide negative contrast enhancement of the target lesion [37, 38]. The *T*_2_ contrast imaging capabilities of Her2-GPH NPs with various Fe concentrations were evaluated at a 0.5 T magnetic field. Figure 10 a displayed the transverse relaxation rates (1/*T*_2_) of water protons as a function of the iron concentration in Her2-GPH NPs aqueous solution. And the relaxivity (*r*_2_) was calculated based on the slope to be 441.47 mM^−1^ s^−1^, which is more than 2 times as high as commercial SPIO-based MR imaging contrast agent Feridex (152 mM^−1^ s^−1^) under the same magnetic field strength [39]. The higher *T*_2_ relaxivity may be due to the fact that the aggregation of SPIOs inside the polymeric matrix results in a relatively enlarge in the size of the magnetic NPs and enhances the magnetic interactions [37, 40]. In addition, the signal intensity of *T*_2_-weighted MR imaging gradually decreased along with the increase of the iron concentration (Fig. 10b), which further confirmed Her2-GPH NPs had the capability for *T*_2_-weighted MR contrast imaging.

#### Targeted MR Imaging of Breast Cancer Cells

The targeted MR imaging effect of Her2-GPH NPs on SKBR3 and MDA-MB-231 cells was confirmed by in vitro *T*_2_-weighted MR imaging. As shown in Fig. 10 c and d, SKBR3 cells treated with targeted Her2-GPH NPs exhibited noticeable negative contrast enhancement, and the signal intensity decreased by 48% compared to the control cells (*P* < 0.001). However, the MR signal intensity of non-targeted GPH NPs-treated SKBR3 cells showed little reduction. In addition, the signal intensity of MDA-MB-231 cells labeled with Her2-GPH NPs or GPH NPs did not change significantly compared with the control group (*P* > 0.05). Furthermore, Her2-GPH NPs-treated SKBR3 cells showed a higher degree of signal intensity reduction than that of MDA-MB-231 cells (*P* < 0.001), which could be used to distinguish the two cells from each other. The above results together indicated that Her2-GPH NPs could enhance *T*_2_-weighted MR imaging through receptor-mediated cell-specific uptake [41].

### In Vitro Targeted PTT Evaluation

**Fig. 11:**
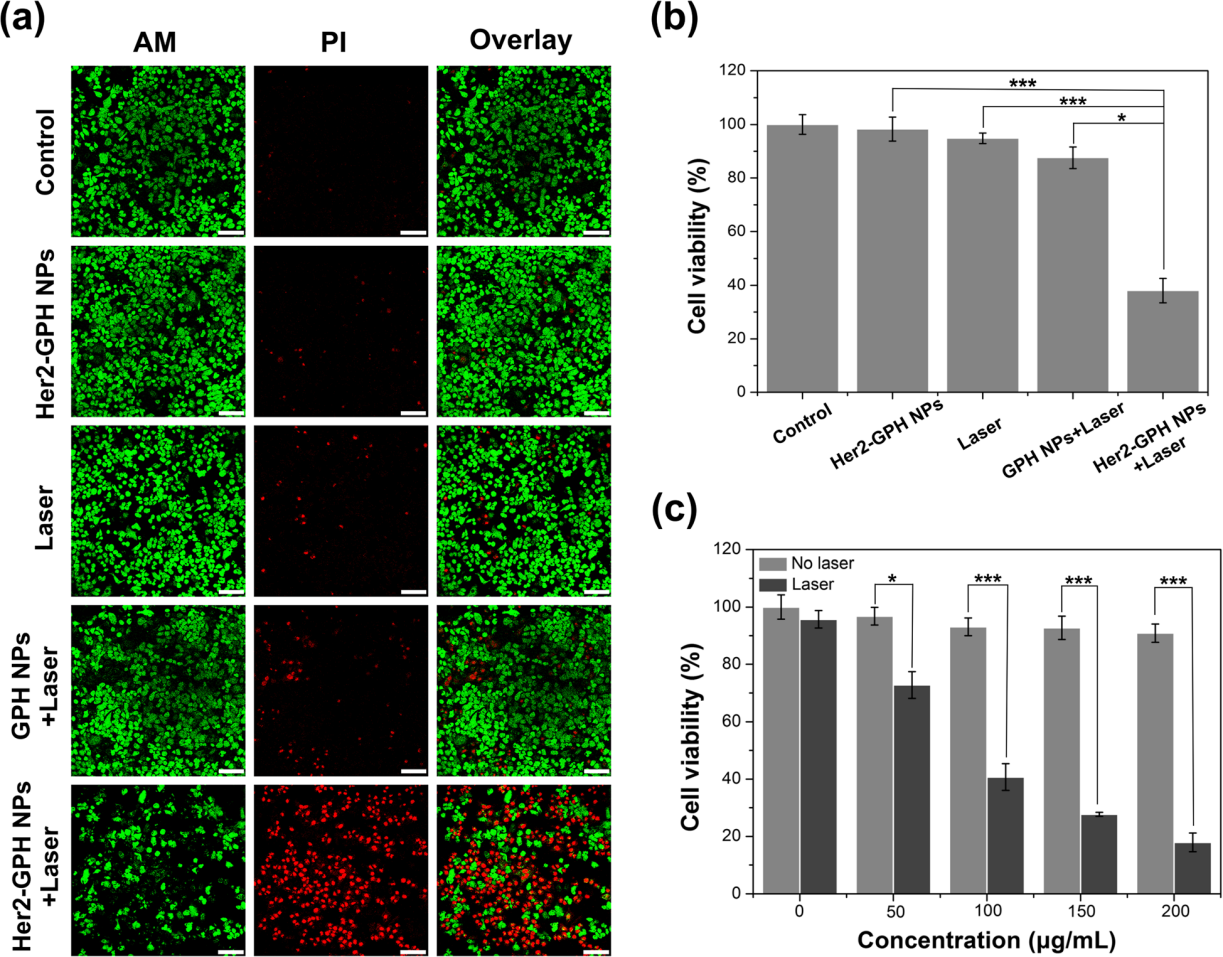
In vitro targeted photothermal assay. **a** Confocal microscopic images of calcein-AM/PI-stained SKBR3 cells in in different treatments groups (Her2-GPH NPs group, laser group, GPH NPs + laser group, Her2-GPH NPs + laser group) (scale bar = 100 μm). SKBR3 cells served as control. **b** Relative cell viabilities of SKBR3 cells in the five groups after treatment as shown in Fig. 11 **a** (data expressed as mean ± SD, *P < 0.05, ***P < 0.001, *n* = 4); **c** cell viabilities of SKBR3 cells incubated with Her2-GPH NPs at different concentrations (0, 50, 100, 150, 200 μg/mL) with or without NIR laser irradiation (808 nm, 1 W/cm^2^, 10 min) (data expressed as mean ± SD, *P < 0.05, ***P < 0.001, *n* = 4)

Initially, calcein-AM/propidium iodide (PI) staining was used to visually evaluate targeted photothermal cytotoxicity in vitro. Calcein AM, a non-fluorescent live cell stain, can permeate the cell membrane and hydrolyze to produce a green fluorescent calcein dye in live cells. Propidium iodide (PI) is a dead cell stain, which binds to the DNA of dead cells to generate red fluorescence [33]. As shown in Fig. 11a, there were no obvious dead cells when NIR laser irradiation or the Her2-GPH NPs was used separately, indicating that neither laser irradiation nor the NPs itself was cytotoxic. Likewise, cells treated with non-targeted GPH NPs in combination with NIR laser showed little cell death. On the contrary, a large number of dead cells could be observed when Her2-GPH NPs were treated in the presence of laser irradiation, which suggested that the agent could induce hyperthermic cell death by targeting photothermal effect. The cell viabilities were further quantitatively evaluated by CCK-8 assay. As illustrated in Fig. 11b, SKBR3 cells treated with Her2-GPH NPs and laser irradiation exhibited a (62 ± 4.56%) decrease in cell viability compared with the control group (*P* < 0.001). Furthermore, under the laser irradiation for 10 min, the cell viability of targeted Her2-GPH NPs group was significantly lower than that of the non-targeted group (38 ± 4.56% vs 87.6 ± 4.06%, *P* < 0.05).

In addition, we used the CCK-8 assay to further quantitatively assess the photothermal cytotoxicity of Her2-GPH NPs at various concentrations. As shown in Fig. 11c, the viabilities of SKBR3 cells treated with Her2-GPH NPs without NIR laser irradiation remained more than 90%, whereas the viabilities of laser irradiated cells decreased significantly with increasing concentrations of Her2-GPH NPs. Only less than 20% of laser-irradiated cells remained viable at the concentration of 200 μg/mL compared with the non-laser-irradiated cells at the same concentration (*P* < 0.001). These results further confirmed that relatively low concentrations of Her2-GPH NPs were sufficient to provide enough temperature increase to kill SKBR3 cells.

Furthermore, we co-cultured SKBR3 and MDA-MB-231 cells in different proportions to simulate the heterogeneous expression of Her2 in breast tumor tissues. Results showed that the total cell viabilities after photothermal induction of Her2-GPH NPs decreased with the increasing proportions of SKBR3 cells, and the total cell viability of the other proportion group was significantly higher than that of the 100% SKBR3 cells group (*P* < 0.05) (Additional file 1: Figure S1). Besides, in the presence of Her2-GPH NPs for 30 min, the total cell viabilities after induction with NIR laser was significantly lower than that of the non-laser irradiation cells (*P* < 0.05), except for the 100% MDA-MB-231 cells group. Taken together, Her2-GPH NPs could specifically bind to SKBR3 cells and had great potential to induce cancer cells to death by photothermal effect. The possible mechanism of photothermal hyperthermia for SKBR3 cells should be mainly attributed to the exposure of the cancer cells to higher temperatures, which inhibited normal cellular growth and proliferation by denaturing intracellular proteins [42].

## Conclusions

Her2-GPH NPs, a Her2 functionalized theranostic agent that integrated dual-modal US/MR imaging and targeted PTT has been successfully designed, fabricated and investigated in vitro. By conjugation with anti-Her2 antibodies, the agent could recognize or bind to breast cancer SKBR3 cells overexpressing Her2 with high specificity and sensitivity. In addition, through the co-loading of PFOB and SPIOs, and the construction of Au-nanoshelled PLGA “shell-core structure”, the resulting Her2-GPH NPs provide excellent contrast enhancement in vitro US and *T*_*2*_-weighted MR imaging. Furthermore, the formation of Au nanoshells enables the agent to be served as efficient photoabsorber for targeted NIR PTT in breast cancer cells. Our results provide a preliminary exploratory study for the integration of collaborative diagnosis and adjuvant therapy of breast cancer, and further studies in vivo are still needed.

## Additional file

Additional file 1: **Figure S1.** Total cell viabilities of co-cultured SKBR3 and MDA-MB-231 cells in different proportions. (TIF 308 kb)

## Data Availability

The datasets generated and analyzed during the current study are included in this article.

## Abbreviations

Au NPs: Gold nanoparticles
CCK-8: Cell-counting kit-8
DAPI: 4′,6-Diamidino-2-phenylindole
DI: Deionized
DLS: Dynamic laser scattering
DMEM: Dulbecco’s modified Eagle’s medium
EDC: Ethyl-3-(3-dimethylaminopropyl) carbodiimide
EDS: Energy dispersive X-ray spectroscopy
FCM: Flow cytometry
FDA: Food and Drug Administration
FE-SEM: Field emission scanning electron microscopy
FITC: Fluorescein isothiocyanate
GPH NPs: Gold-nanoshelled magnetic hybrid nanoparticles
Her2: Human epidermal growth factor receptor 2
HR-TEM (TEM): High-resolution transmission electron microscope
HUVEC cells: Human umbilical vein endothelial cells
ICP-AES: Inductively coupled plasma atomic emission spectroscopy
LSCM: Laser scanning confocal microscopy
MRI: Magnetic resonance imaging
NIR: Near-infrared
PAH: Polyallylamine hydrochloride
PFOB: Perfluorooctyl bromide
PLGA: Poly lactic-co-glycolic acid
PTT: Photothermal therapy
PVA: Polyvinyl alcohol
SPIOs: Superparamagnetic iron oxide nanoparticles
TIC: Time-intensity curve
UCA: Ultrasound contrast agent
US: Ultrasound imaging
