# Supplementary figures and images for: Her2-Functionalized Gold-Nanoshelled Magnetic Hybrid Nanoparticles: a Theranostic Agent for Dual-Modal Imaging and Photothermal Therapy of Breast Cancer
Source: Nanoscale Res Lett. 2019 Aug 26;14:235. doi: 10.1186/s11671-019-3053-4 (PMC6709082; doi:10.1186/s11671-019-3053-4)

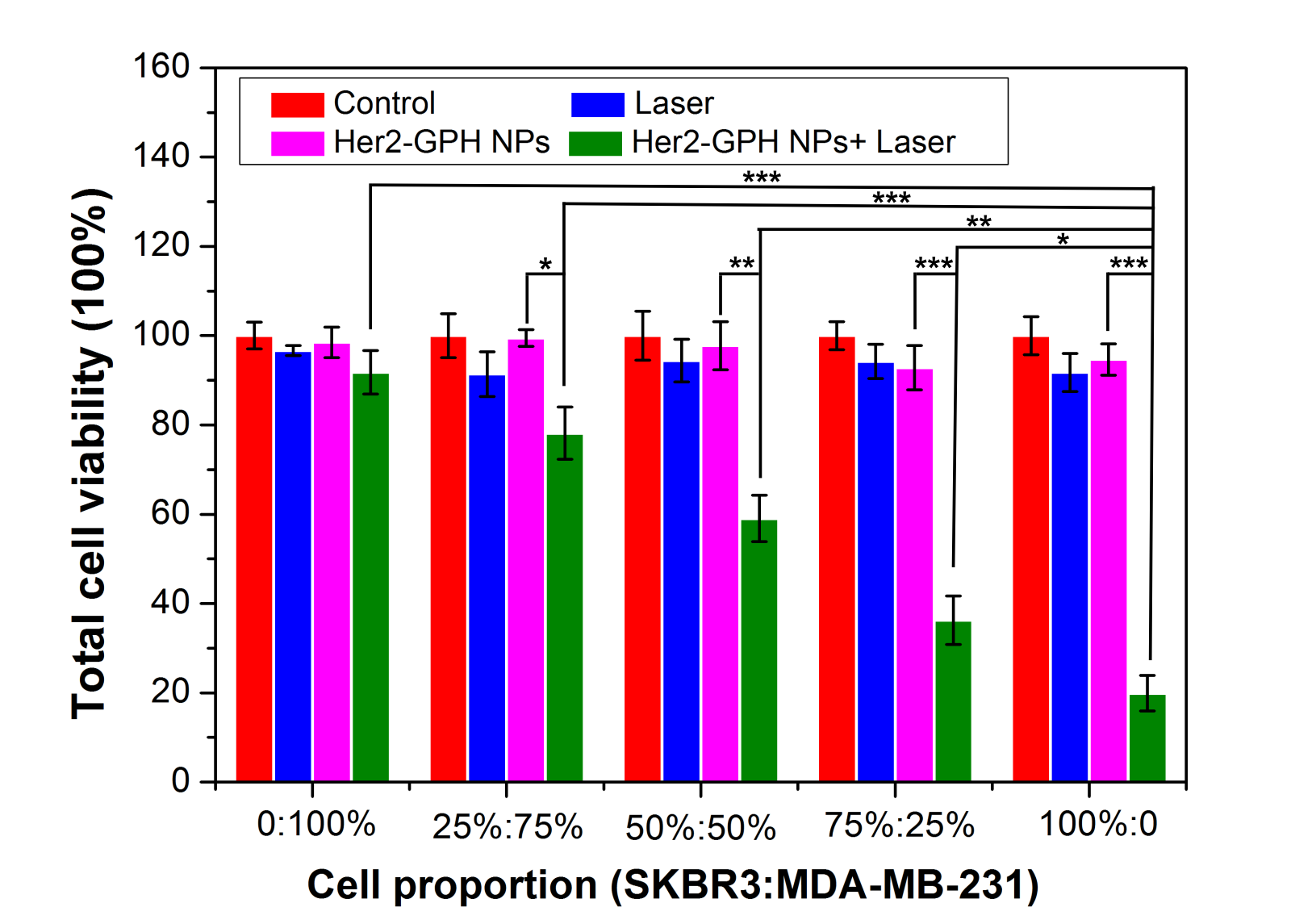

Supplement: Supplementary file 1 — Figure S1. Total cell viabilities of co-cultured SKBR3 and MDA-MB-231 cells in different proportions. (TIF 308 kb) [file 11671_2019_3053_MOESM1_ESM.tif]
